# Comparative analysis of mammalian sperm ultrastructure reveals relationships between sperm morphology, mitochondrial functions and motility

**DOI:** 10.1186/s12958-019-0510-y

**Published:** 2019-08-15

**Authors:** Ni-Hao Gu, Wen-Long Zhao, Gui-Shuan Wang, Fei Sun

**Affiliations:** 1 International Peace Maternity and Child Health Hospital, School of Medicine,Shanghai Jiao Tong University, Shanghai, 200030 China; 2 Shanghai Key Laboratory of Embryo Orignal Diseases, Shanghai, 200030 China; 3 Shanghai Municipal Key Clinical Speciality, Shanghai Jiao Tong University, Shanghai, 200030 China; 4 Institute of Reproductive Medicine, School of Medicine, Nantong University, Nantong, 226001 Jiangsu China

**Keywords:** Axoneme, Outer dense fibers, Mitochondrial sheath, Fibrous sheath, Sperm motility, Mitochondrial function, Flagellar lengths

## Abstract

**Background:**

Sperm morphology mainly refers to the shape of the head, the length of the flagellar segments, including the midpiece, principal piece and end piece, and the size of the accessory structures, including axonemes, outer dense fibers (ODFs), mitochondrial sheath (MS) and fibrous sheath (FS). Across species, there is considerable diversity in morphology. An established theory posits that the length of the sperm flagellum, especially the length of the midpiece, is a critical factor influencing sperm metabolism and velocity. However, our understanding of the relationships between sperm ultrastructures and the sperm flagellar length is incomplete.

**Methods:**

The morphologies of sperm from 10 mammalian species, human, mouse, rat, dog, rabbit, goat, pig, bull, guinea pig and golden hamster, were examined by scanning electron microscopy (SEM) and transmission electron microscopy (TEM). According to the SEM and TME images, the length of sperm heads and flagellar segments, the cross-sectional areas of the accessory structures and flagella and the width of sperm heads were measured using Image J software. The variation tendencies (referred to as slope) of the accessory structures along flagella were calculated by the linear regression method. Mitochondrial functions were measured using commercial kits. The velocities of sperm were measured using CASA software.

**Results:**

The three-dimensional morphologies of sperm from 10 species and the slopes of internal accessory structures along flagella were obtained. The width of the axoneme tapered slightly from the base to the tip of the sperm flagellum, and slopes of the axonemes correlated negatively with the variability in flagellar length across species. Additionally, the cross-sectional areas of the ODFs and/or the MS were positively correlated with the lengths of the midpiece, principal piece, and total flagellum, as well as with sperm velocities. Mitochondrial volumes were positively correlated with ATP content and sperm swimming velocities.

**Conclusions:**

Our results not only show the relationship between sperm internal structures, flagellar length and sperm physiology but also provide sizes of mitochondria and ODFs as new targets with which to study the regulation of sperm length and velocity.

**Electronic supplementary material:**

The online version of this article (10.1186/s12958-019-0510-y) contains supplementary material, which is available to authorized users.

## Background

Morphologic sperm traits, including the shape of the head and the length of the flagellum, are diverse in mammals. In general, the width and length of the sperm head vary due to variations in the size and organization of the acrosome and nuclei [1]. Alternatively, the sperm flagellum can be divided into the following four distinct segments: the connecting piece, also termed the neck; the middle piece, or midpiece; the principal piece; and the end piece [2]. The connecting piece is adjacent to the head and contains degenerated centrioles, which lack many structural characteristics and protein composition in comparison with typical centrioles in somatic cells [3], and transitional structures linked with internal structures in the midpiece [4]. The midpiece is defined by the cytoskeletal structures surrounded by packed mitochondria [5]. The principal piece features the fibrous sheath (FS) that surrounds the cytoskeleton [6]. The end piece is a prolongation of the principal piece without any accessory structures [7]. The lengths of the connecting piece and the end piece are usually short across species, whereas the lengths of the midpiece and the principal piece contribute to the variation among sperm flagella in mammals [8]. The mitochondria are the triphosphate adenosine (ATP) source in the sperm midpiece and produce and transport energy to the axoneme in the principal piece, which generates the driving force of the sperm [9]. Thus, the lengths of the sperm midpiece and principal piece may be critical in determining sperm swimming velocity and the rate of fertilization [10–13].

In addition to large variations in the shape of the head and the length of the flagellum, the morphology of the internal ultrastructures containing the mitochondrial sheath (MS), outer dense fibers (ODFs) and fibrous sheath (FS) differ widely between mammalian species [14, 15]; there is little variation in axonemes between species. The axoneme exhibits a conserved 9 + 2 microtubule arrangement in cross-section, with nine doublet microtubules linking to a central pair complex that is composed of two singlet microtubules. The coordinated activities of multiple dynein are driven by hydrolysing ATP in the axoneme to drive flagellar motility [16]. Traditionally, it is thought that the width of the axoneme is constant from the base to the tip of the sperm flagella, even across species [17].

The MS defines the region of the midpiece and surrounds the ODFs and axoneme. The MS consists of different numbers of mitochondria, which are elongated and arranged in a helix pattern, called gyres, from species to species. For example, there are an estimated 10–12 gyres in humans, 15–17 in dogs, 97 in mice and 350 in rats [14].

Ordinarily, the ODFs comprise a set of nine striated columns that start in the connecting piece and end at a specific position of the tail in certain species. In longitudinal view, each fiber is largest at the proximal end of the midpiece and tapers progressively along the flagellum, with each ending at different positions along the principal piece [18]. Cross-sectionally, the shape and size of each fiber differ between and within species. Fibers 1, 5, 6, and sometimes 9, namely odfs 1, 5, 6, and 9, are generally larger than odfs 2, 3, 7 and 8 [19]. It is thought that the ODFs not only protect the tail from damage caused by the shearing force during epididymal transport and ejaculation but also increase the stiffness of the flagellum and stabilize the axoneme [20, 21].

The FS is a cytoskeletal structure of the principal piece. It consists of two longitudinal columns connected by semicircular ribs. The size of the longitudinal columns is largest at the anterior part of the principal piece and decreases gradually along the principal piece [22]. Cross-sectionally, the shape and size of the longitudinal columns, but not the semicircular ribs, exhibit major variance across mammalian species. In the present study, the area of the FS is represented by the cross-sectional area of the longitudinal columns. It is believed that the FS serves as a scaffold for proteins participating in glycolysis, cAMP-dependent signalling transduction and mechanical support [23, 24].

Although these structures of the flagellum have been characterized in many species, limited data about the three-dimensional nature of these internal structures between and within species have been reported. Furthermore, the relationships between the three-dimensional internal ultrastructures and sperm morphology, as well as sperm swimming velocity and mitochondrial functions, have been poorly explored.

To address these questions, sperm from ten common species, including guinea pig, mouse, rat, golden hamster, dog, bull, pig, rabbit, goat and human, were collected and analysed by transmission electron microscope (TEM) and scanning electron microscope (SEM) combined with biochemical assays and previous swimming velocity data. Our study not only provides quantitative data to describe differences in the three-dimensional ultrastructures of flagella and sperm morphologic characteristics but also resolves the relationships mentioned above.

## Materials and methods

### Sperm collection

Sperm samples from rat, mouse, guinea pig, golden hamster and rabbit were collected from the cauda epididymis, as described previously [21]. Briefly, mature animals were purchased from SLAC Laboratory Animal Co., Ltd. (Shanghai, China). The cauda epididymis were removed from euthanized animals and dissected. The sperm swam from tissues submerged in 500 μl of Tyrode’s salt solution (T2397, Sigma, USA) at 37 °C in a humidified 5% CO_2_ atmosphere for 15 min. Next, the sperm were harvested by centrifugation at 150 g for 5 min at room temperature and were prepared for TEM, SEM, or biochemical analysis.

Ejaculated human and dog sperm were collected using the gloved-hand technique. The human semen samples, including 46 normozoospermic and 25 asthenozoospermic samples, were obtained from the International Peace Maternity & Child Health Hospital (IPMCH). The details of the human samples, including the sperm semen volume, sperm count, motility and so on, are listed in Additional file 14: Table S6. The dog semen samples were a gift from the School of Agriculture and Biology, Shanghai Jiao Tong University. The liquefied semen was washed in a 10-volume excess of Tyrode’s solution. The sperm were harvested by centrifuging at 150 g for 5 min.

The goat, bovine and pig semen were purchased from Yuan Du Husbandry Co., Ltd. (Shanghai, China), Shang Mi Agriculture and Technology Co., Ltd. (Beijing, China) and Shanghai Engineering Research Center of Pig Breeding of Sun Sing Co., Ltd. (Shanghai, China), respectively. Ejaculated goat, bovine and pig sperm samples were collected using an artificial vagina as described previously [25] and then shipped frozen to our laboratory. Prior to use, the sperm were washed twice in a 10-volume excess of Tyrode’s solution to isolate the semen. Next, sperm were collected by centrifugation at 150×g for 5 min.

### Scanning electron microscopy

The sperm from 3 males per species were plated on poly-D-lysine-coated cover glasses (diameter of 12 mm). Then, the cover glasses were submerged into fixation solution (2.5% glutaraldehyde in 0.1 M HEPES buffer, pH = 7.5) to fix the sperm overnight at 4 °C. Next, the sperm were post-fixed in 1% osmium tetroxide in 0.1 M HEPES buffer (pH = 7.5) and were sequentially dehydrated in 30, 50, 70, 80, 95, 100, and 100% ethanol solutions for 10 min each. Sperm were then processed as previously described [26] and subsequently examined in the scanning electron microscope (Quanta 250, FEI Co., Ltd., USA) at 2000 × and 20,000 × magnification, excluding the human sample, which was examined at 3000 × and 20,000 × magnification.

### Transmission electron microscopy

Sperm from 3 males per species were suspended in the fixation solution overnight at 4 °C and processed by the electron microscopy service centre at Shanghai Jiao Tong University of Medicine following a conventional protocol. The samples were observed via electron microscopy (H-7650, HITACHI, Japan) at 15,000 × magnification.

### Morphometric data acquisition and data analysis

Data on mammalian sperm straight line velocity (μm/s) (VSL: velocity calculated using the straight-line distance between the beginning and end of the sperm track) were acquired using computer-assisted sperm analysis software (Hamilton Thorne, Beverly, MA, USA).

**Fig. 1:**
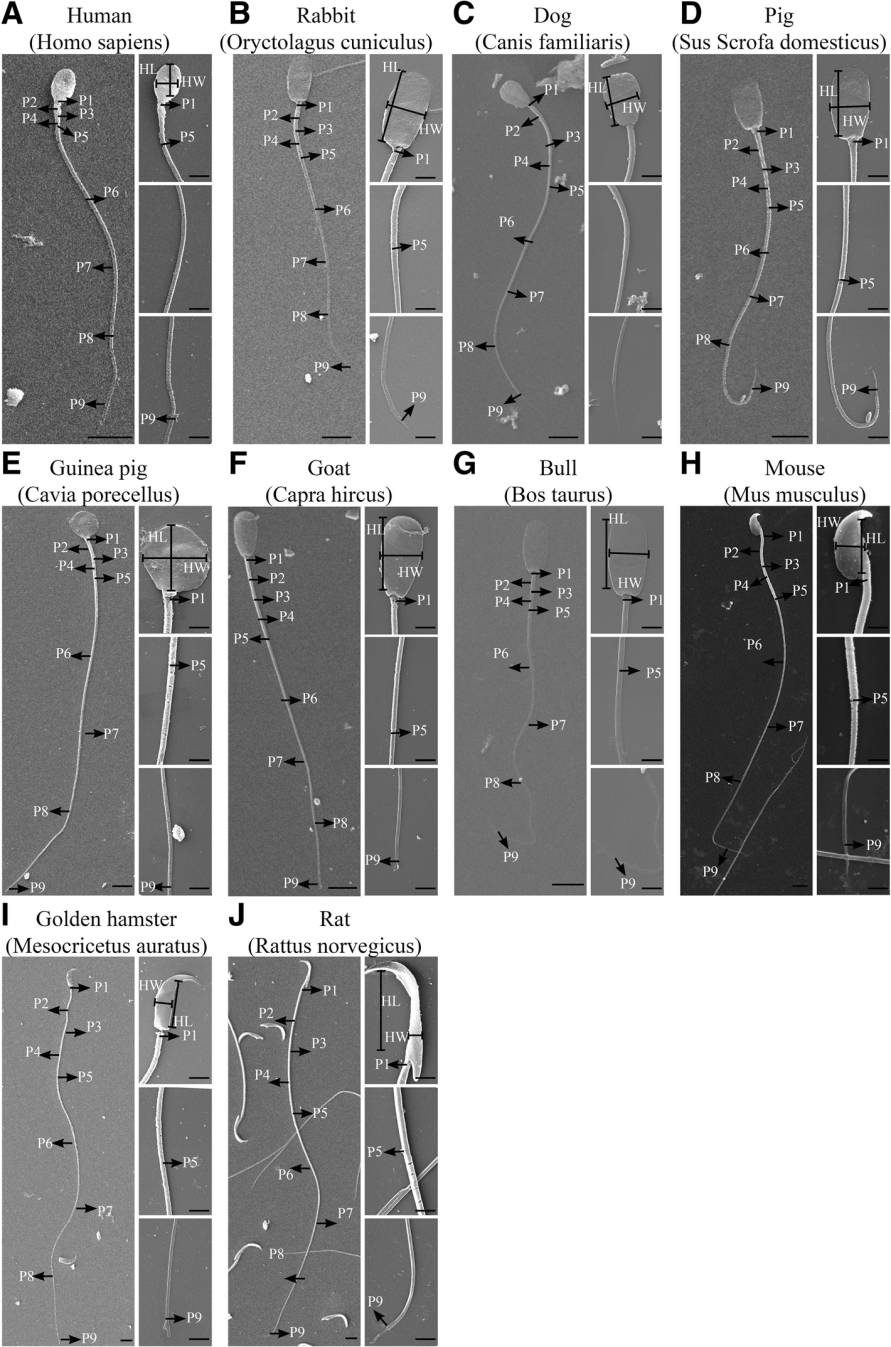
Scanning electron micrographs of sperm from 10 species. **a** Human (*Homo sapiens*). **b** Rabbit (*Oryctolagus cuniculus*). **c** Dog (*Canis familiaris*). **d** Pig (*Sus scrofa domesticus*). **e** Guinea pig (*Cavia porecellus*). **f** Goat (*Capra hircus*). **g** Bull (*Bos taurus*). **h** Mouse (*Mus musculus*). **i** Golden hamster (*Mesocricetus auratus*). **j** Rat (*Rattus norvegicus*). In each panel, the left image shows sperm in whole-view, and the right images sequentially show the head, boundary of the midpiece-principal piece and the terminal of the principal piece in enlarged view. The scale bars are 5 μm in the left image and 2 μm in the right images. Please note the different lengths of the scale bars across the 10 species. The morphological features of the head and flagella are labelled by lines and arrows, respectively (*n* = 30 sperm/male, *n* = 3 males/species). The widths of positions 1–9 were measured as the diameters of flagella in these regions

All images from the SEM and TEM assays were processed by NIH ImageJ. For morphological data of each sperm from the 10 species, sperm head length, head width, midpiece length, principal piece length, end piece length and diameter of 1–9 sites (short for D) on the sperm were measured by the segmented line tool. Sperm head length was defined as the longest region of the sperm head. Sperm head width was defined as the widest region of the head perpendicular to the length measurement. Midpiece length was defined as the region from the base of the sperm head to the boundary of the midpiece-principal piece. Principal piece length was defined as the region from the boundary of the midpiece-principal piece to the principal piece-end piece boundary. End piece length was measured from the end tip of the principal piece to the visible terminal region of the sperm flagella. The flagellum: head ratio was calculated as the flagellar length divided by the head length per sperm cell. These 6 sperm traits from sperm from 10 species are indicated by black lines in Fig. 1 A-J. The means of the 6 sperm traits are shown in Additional file 9: Table S1.

Moreover, positions 1, 5, and 9 were defined as the base of the midpiece (commonly the thickest region of the sperm flagella from 10 species), the boundary of the midpiece-principal piece and the terminal of the principal piece, respectively. Positions 2, 3, and 4 were four sequential, equal diversion points along the midpiece. Meanwhile, positions 6, 7, and 8 were four equal diversion points in the principal piece. The areas of the base of the midpiece and the boundary of the midpiece-principal piece were calculated the mean areas of positions 1 and 5 by using the formula S_outer area_ = (D/2)^2^ × π. All measured regions in sperm from the 10 species are indicated by black arrows in Fig. 1. The mean diameters of the 9 sites along sperm are shown in Additional file 10: Table S2. A minimum of thirty sperm each from 3 males per species were measured via SEM assay.

**Fig. 2:**
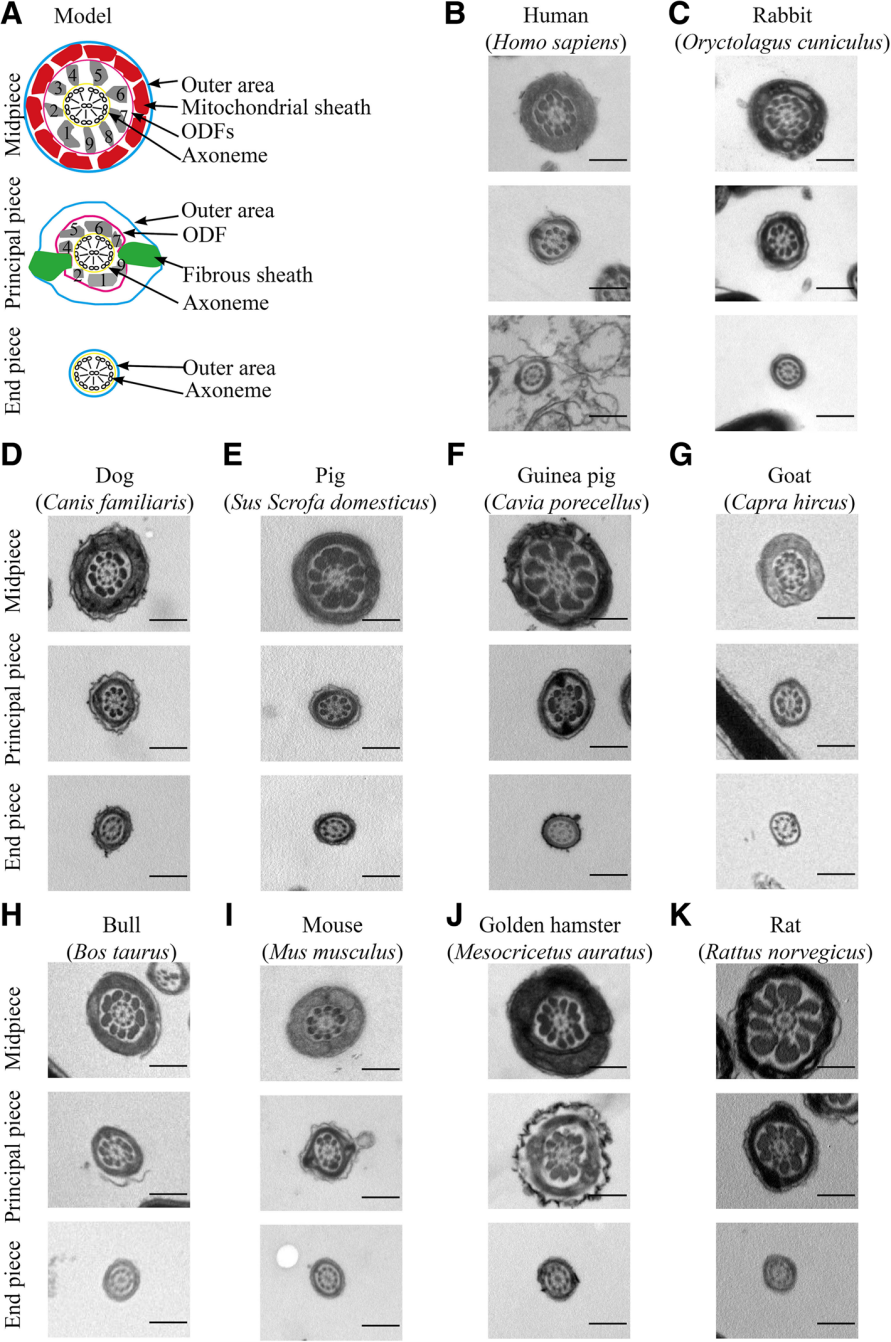
Transmission electron micrographs of flagellar segments from 10 species in cross-sectional view. **a** Diagram detailing key ultrastructures, including axoneme, ODFs, fibrous sheath, and mitochondria (red modes), in three major structural domains. The outer area is indicated as the area surrounded by the blue line; the ODF area is indicated as the area around by the purple line; the area of the axoneme is indicated as the area around by the yellow line; and the green mode indicates the FS area in the principal piece. The numbers show the numbers of odf fibers 1–9. **b** Human. **c** Rabbit. **d** Dog. **e** Pig. **f** Guinea pig. **g** Goat. **h** Bull. **i** Mouse. **j** Golden hamster. **k** Rat. The measured areas were labelled by coloured lines, as shown in panel J. The number of measured segments was more than 30 in each species. The scale bars are 400 nm

To quantify the areas of the sperm ultrastructures, we measured outer areas, mitochondrial areas, ODF areas, axoneme areas, FS areas and odfs 1–9, which are illustrated cross-sectionally in Fig. 2 a. The t-odfs area is indicated as the sum of odfs 1–9 in each cross-section. The typical cases for each species are shown in Fig. 2 b-k. All measured regions in cross-sectional images were measured using the polygon selection tool in NIH/ImageJ, and original data from the areas of these ultrastructures are shown on scatter plots in Additional file 1: Figure S1, Additional file 2: Figure S2, Additional file 3: Figure S3, Additional file 4: Figure S4 and Additional file 5: Figure S5. Linear models were used to test the associations between variation in the areas of the ultrastructures and the outer areas. The area of the ultrastructures was calculated using the equation S_ultrastructures_ = a_Slope_ × S_outer area_ + b for a given outer area along the flagellum. The number of cross-sections per flagellar segment was more than 30 each from 3 males per species. Using the formulas acquired from the TEM assay, we conveniently calculated the mean areas of the ultrastructure relative to positions 1–9 in sperm (Additional file 11: Table S3) and plotted them in Additional file 6: Figure S6 A-F and Additional file 7: Figure S7 A-I.

To determine the mitochondrial volume, we assumed the mitochondrial volume as a fistular frustum of a cone in the midpiece whose volume is V_mito_ = V_mid_-V_ODF_; the midpiece volume and ODF volume were also calculated as the frustums of cones. The midpiece volume is V_mid_ = {S_base-mid_ + (S_base-mid_ × S_tip-mid_)^0.5^ + S_tip-mid_} × L_m_/3, and the ODF volume in the midpiece is V_ODF_ = {S_base-ODF_+(S_base-ODF_ × S_tip-ODF_)^0.5^ + S_tip-ODF_} × L_m_/3. The mitochondrial volumes from each species were listed in Additional file 12: Table S4.

### Measurement of mitochondrial functions

In this study, ATP content and mitochondrial superoxide (mROS) content in sperm samples were determined using an ATP assay kit (S0026, Beyotime, Wuhan, China), MitoSOX red probe (M36008, Thermo Fisher, USA) respectively, to reflect mitochondrial functions. The sperm concentration was adjusted to 2.5 × 10^6^ sperm/ml for ATP content assay. Then, the sperm precipitates were re-suspended in lysis solution at 4 °C. The suspension was collected after centrifugation at 12,000×g for 5 min at 4 °C. The samples were measured in triplicate in 96-well plates using a luminometer (Synergy h1 hybrid microplate reader, BioTek, USA). The signal intensities of MitoSOX were analysed on a FACS Calibur flow cytometer following a previous study [27]. We calculated the mean ATP concentration and mean intensities of mROS from 3 males for each species. The data are listed in Additional file 12: Table S4.

### Statistics

**Fig. 3:**
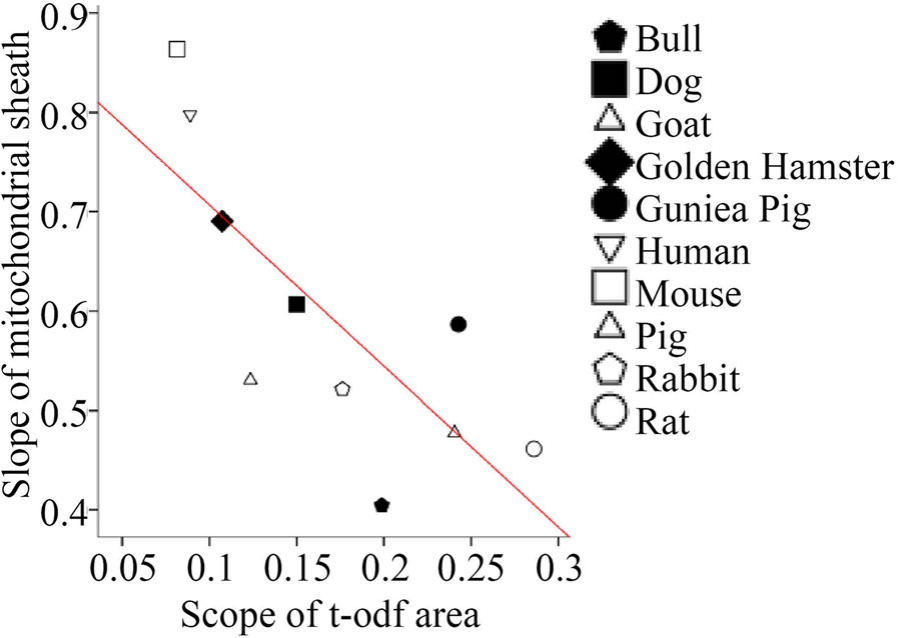
Relationships between the slopes of mitochondria and t-odfs across species. Regression analysis shows a negative relationship between mitochondria and t-odfs (R^2^ = 0.598, *p* < 0.05)

**Fig. 4:**
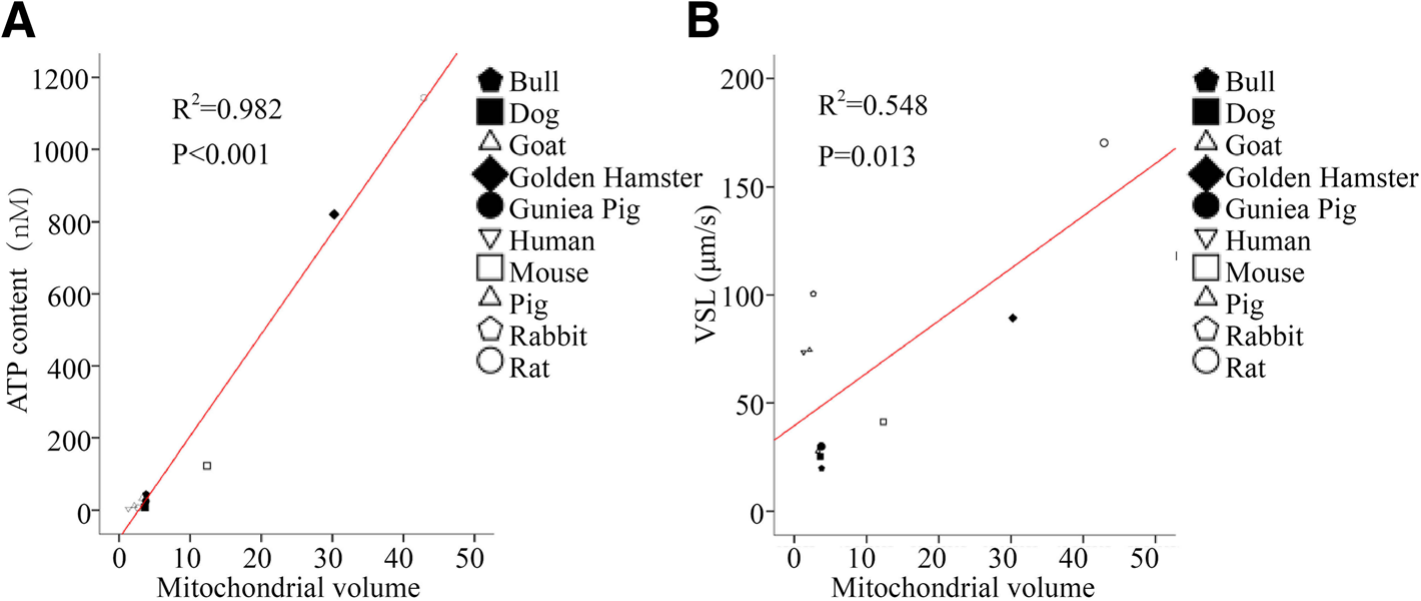
Relationships between mitochondrial volume and ATP content, VSL. **a** Regression analysis shows a positive relationship between mitochondrial volume and ATP content (n = 3 replicates/sample, *N* = 3 male samples/species) (R^2^ = 0.982, *P* < 0.001). **b** Regression analysis shows a positive relationship between mitochondrial volume and VSL (R^2^ = 0.528, *P* = 0.017)

Linear regression was performed to evaluate associations between parameters of the sperm ultrastructures and sperm morphologies. The regression analyses in Figs. 3 and 4 were performed with SPSS 12.0 software, with other analyses and graphics produced by GraphPad Prism 5.0 software. *P* values < 0.05 were considered statistically significant.

## Results

### Quantitative description of slopes of sperm ultrastructures in cross-section

After acquiring the slopes of areas of the internal structures along sperm flagella, regression correlation analyses between outer areas and areas of mitochondria, ODFs, axonemes, FS, odfs 1–9 and length of flagellar segments were performed, and the results are shown in Additional file 13: Table S5. As expected, the cross-sectional areas of mitochondria, ODFs, FS, odfs 1–9 and t-odfs tapered progressively along flagella in all 10 species (Additional file 1: Figure S1, Additional file 2: Figure S2 and Additional file 3: Figure S3), consistent with previous studies. The slope of odf1 was the largest, while the slopes of odf5 and odf6 were the next largest and generally exhibited similar patterns in each of the ten species. Moreover, the slopes of odfs 2, 3, 4, 7, 8, and 9 converged in each of the ten species (Additional file 4: Figure S4 A-J). Empirically, the areas of odfs 2, 3, 4, 7, 8, and 9 disappeared earlier than those of odfs 1, 5, and 6. Notably, the area of the axoneme also tapered along the sperm flagellum in each species, contrary to the traditional opinion that the widths of the axonemes do not vary in different segments of the sperm flagellum, even across species (Additional file 5: Figure S5).

### Relationships between sperm traits and slopes of sperm ultrastructures

We first examined the association between slopes of these ultrastructures and found that the slopes of the mitochondrial areas were negatively correlated with the slopes of the t-odf areas (R^2^ = 0.598, *p* < 0.05) (Fig. 3) and odfs 1–9 (Table 1) in each of the 10 species. Moreover, the slopes of odfs 1–9 were positively correlated with each other (Table 1). All correlation coefficients and *p*-values between the slopes of these ultrastructures are shown in Table 1.

**Table 1:** Correlations between slopes of the sperm ultrastructures across 10 species

Ultrastructure name	ODFs	Axonome	FS	MS	odf1	odf2	odf3	odf4	odf5	odf6	odf7	odf8	odf9	t-odf area
Axonome	−0.07													
FS	0.11	0.51												
MS	−0.79^**^	−0.32	− 0.27											
odf1	0.98^***^	0.16	0.04	−0.87^***^										
odf2	0.97^***^	0.04	−0.14	−0.78^**^	0.95^***^									
odf3	0.91^***^	0.04	0.05	− 0.92^**^	0.94^***^	0.92^***^								
odf4	0.98^***^	0.04	−0.14	− 0.78^**^	0.94^***^	0.97^***^	0.93^***^							
odf5	0.99^***^	0.1	−0.02	− 0.82^**^	0.99^***^	0.96^***^	0.92^***^	0.95^***^						
odf6	0.99^***^	0.11	−0.01	− 0.83^**^	0.99^***^	0.97^***^	0.93^***^	0.96^***^	1^***^					
odf7	0.95^***^	0	−0.09	− 0.81^**^	0.92^***^	0.98^***^	0.95^***^	0.98^***^	0.93^***^	0.94^***^				
odf8	0.82^**^	0.08	0.07	−0.89^**^	0.88^***^	0.87^***^	0.94^***^	0.83^***^	0.86^***^	0.87^***^	0.89^***^			
odf9	0.96^***^	0.12	−0.16	−0.74^*^	0.92^***^	0.99^***^	0.88^***^	0.96^***^	0.93^***^	0.94^***^	0.96^***^	0.8^**^		
t-odf area	0.84^**^	−0.22	−0.08	−0.65^*^	0.81^***^	0.89^***^	0.83^***^	0.86^***^	0.83^***^	0.85^**^	0.92^***^	0.81^**^	0.85^**^	

*P<0.05, **P<0.01, ***P<0.001. Analyses with autocorrelation between variables were not included in the table.t-odf area = (odf1–9) total area

Second, we found that the slopes of axonemes were negatively correlated with four sperm flagellar traits (midpiece length, principal piece length, total flagellar length and flagellum: head ratio). However, there were no significant correlations between the mean of the sperm traits and other slopes of the ultrastructures (Table 2).

**Table 2:** Correlations between slopes of the sperm ultrastructures and the sperm morphology

Sperm morphology Ultrastructures	Head length	Head width	Midpiece length	Principal length	Flagellar length	Flagellum:head ratio
ODFs	0.37	0.16	0.18	−0.01	0.07	− 0.06
Axonome	−0.15	0.24	−0.70^*^	− 0.78^**^	−0.78^**^	− 0.72^*^
Fibrous sheath	0.14	0.06	−0.44	−0.63	− 0.58	−0.59
Mitochondrial sheath	−0.18	−0.02	− 0.01	0.28	0.18	0.24
odf1	0.44	0.11	0.20	−0.04	0.06	−0.08
odf2	0.45	0.26	0.15	−0.04	0.04	−0.12
odf3	0.31	0.04	0.20	−0.08	0.03	−0.08
odf4	0.29	0.13	0.19	−0.02	0.06	−0.05
odf5	0.45	0.10	0.25	0.02	0.11	−0.03
odf6	0.45	0.12	0.22	−0.02	0.08	−0.07
odf7	0.36	0.19	0.15	−0.09	0.01	−0.13
odf8	0.45	0.02	0.21	−0.16	−0.02	− 0.17
odf9	0.39	0.36	0.03	−0.10	−0.05	− 0.19
t-odf area	0.56	0.22	0.27	0.02	0.12	−0.07

*P<0.05, **P<0.01. t-odf area = (odf1 + odf2 + odf3 + odf4 + odf5 + odf6 + odf7 + odf8 + odf9) area

### Association between the flagellar lengths and the cross-sectional areas of sperm ultrastructures

We next asked whether the mean cross-sectional areas of the sperm ultrastructures in the base of the midpiece, the boundary of the midpiece-principal piece and the end piece were correlated with the flagellar lengths across the 10 species. Table 3 shows that the mean cross-sectional areas of both ODFs and mitochondria in the base of the midpiece were positively correlated with all three flagellar lengths across 10 species. Similarly, the mean cross-sectional areas of both odfs 1–9 and t-odfs in the base of the midpiece were positively correlated with all three flagellar lengths. However, the mean cross-sectional areas of the axoneme and the FS in the base of the midpiece were not significantly correlated with the flagellar length measurements (Table 3). Furthermore, the cross-sectional areas of both the ODFs and mitochondria, rather than the axoneme or the FS, in both the boundary of the midpiece-principal piece and the terminal of the principal piece were significantly correlated with the flagellar length measurements (data not shown).

**Table 3:** Correlations between cross-sectional areas of the sperm ultrastructures in the base of midpiece and sperm morphology

Sperm morphology Ultrastructures	Head length	Head width	Midpiece length	Principal length	Flagellar length	Flagellum:head ratio
ODFs	0.3379	−0.2861	0.7733^**^	0.5679	0.6755^**^	0.5389
Axoneme	0.3994	0.0832	0.1348	0.0291	0.0676	−0.0368
FS^a^	−0.0256	− 0.0020	0.1376	0.4675	0.3561	0.4017
MS	0.0186	−0.5027	0.8374^**^	0.9286^***^	0.9322^***^	0.9136^***^
odf1	0.3635	−0.2991	0.8^**^	0.5873	0.6981^*^	0.5562
odf2	0.3493	−0.2174	0.7002^*^	0.4812	0.5910	0.4478
odf3	0.3299	−0.2632	0.7396^*^	0.5188	0.6306	0.4943
odf4	0.2942	−0.2750	0.7278^*^	0.5043	0.6169	0.4873
odf5	0.3467	−0.3025	0.7876^**^	0.5694	0.6818^*^	0.5431
odf6	0.3567	−0.2875	0.7745^**^	0.5514	0.6651^*^	0.5227
odf7	0.3020	−0.2633	0.7255^*^	0.5076	0.6182	0.4874
odf8	0.3149	−0.2702	0.7389^*^	0.5183	0.6301	0.4974
odf9	0.3185	−0.1793	0.688^*^	0.5101	0.6057	0.4696
t-odf area	0.3291	−0.2824	0.7627^**^	0.5492	0.6594^*^	0.5234

*P<0.05, **P<0.01, ***P<0.001. Analyses with autocorrelation between variables were not included in the table. a: the areas of FS indicate the areas of FS in the boundary of midpiece and principal piece. .t-odf area = (odf1 + odf2 + odf3 + odf4 + odf5 + odf6 + odf7 + odf8 + odf9) area

### Establishing relationships between the sperm ultrastructures, swimming velocity and mitochondrial functions

Extensive studies have demonstrated that flagellar lengths, especially midpiece length, are good predictors for sperm swimming velocity [10, 28], and it has also been shown that mitochondrial volume determines the flagellar beat frequency [29]. Thus, we addressed whether relationships exist between the mean cross-sectional areas of sperm ultrastructures in the base of the midpiece and the swimming velocity and mitochondrial functional parameters, and we also considered whether a relationship exists between the mitochondrial volume and swimming velocity and ATP content across 10 species. We found that areas of both the ODFs and mitochondria, but not the axoneme or FS, were significantly correlated with the ATP content (Table 4). The odfs 1–9 and t-odfs were also significantly positively associated with the ATP content (Table 4), whereas only the area of the mitochondria was positively correlated with VSL (Table 4).

**Table 4:** Correlations between cross-sectional areas of the sperm ultrastructures in the base of midpiece and sperm physiology

Ultrastructures	VSL	ATP content
ODFs	0.22	0.77^**^
Ax	0.12	0.08
FS^a^	0.39	0.13
MS	0.79^**^	0.78^**^
odf1	0.26	0.8^**^
odf2	0.12	0.7^*^
odf3	0.18	0.74^*^
odf4	0.17	0.73^**^
odf5	0.24	0.79^**^
odf6	0.23	0.77^**^
odf7	0.18	0.72^*^
odf8	0.18	0.74^*^
odf9	0.14	0.68^*^
t-odf area	0.21	0.76^**^

*P<0.05; **P<0.01. Analyses with autocorrelation between variables were not included in the table. a: the areas of FS indicate the areas of FS in the boundary of midpiece and principal piece. t-odf area = (odf1 + odf2 + odf3 + odf4 + odf5 + odf6 + odf7 + odf8 + odf9) area

Furthermore, linear regression analysis revealed positive relationships between mitochondrial volume and the ATP content (R^2^ = 0.982, *P* < 0.001) and VSL (R^2^ = 0.548, *P* = 0.013) (Fig. 4. A, B), but not mROS concentrations (*P* = 0.93). These results suggest the areas of the ODFs, even each odf fiber, are potential predictors of sperm physiology. Mitochondria might be the major source of ATP in sperm, though sperm velocity is to a certain extent determined by factors other than ATP production.

## Discussion

Morphological normality of sperm flagella is critical for sperm motility and fertilization processes. Sperm morphology assessments, including the morphologies of the sperm head, midpiece and other flagellar components, not only reflect the sperm quality and environmental factors during spermiogenesis but also become an important cue for diagnosis for male infertility in the clinic [30, 31]. Additionally, we enhanced our understanding of the details and relationships between sperm micro- and macro-morphologies, which will advance understanding of the underlying mechanisms of sperm motility. For instance, the mitochondrial sheath not only produces optimal levels of ROS and ATP but also prevents the internal structures from buckling out from the flagellum during sperm swimming. ATP is transported to the axoneme and hydrolysed by dyneins, which are distributed in the axoneme asymmetrically and generate an imbalance force to drive flagellar motility [32]. The functions of ODFs and FS are not fully understood. In general, the ODFs are considered as restraining structures that increase the stiffness of the flagellum and amplifiers that increase the bending torque generated by the axoneme’s bending movement [33]. The FS also acts as a rigid structure that increases the stiffness of the flagellum; meanwhile, it is involved in glycolysis and Ca^2+^ signalling transduction dependence with relevant enzymes and ion channels [34–36]. Thus, descriptions of the three dimensions of sperm components, including head length, head width, midpiece length, principal length, flagellar length, areas of internal flagellar ultrastructures in each segment and the volume of mitochondria across 10 common species, may advance knowledge regarding sperm motility.

In this work, relationships between the internal flagellar ultrastructures, the lengths of the flagellar components and sperm motility were established. We also found that the areas of mitochondria and ODFs, as well as the major components of odf fibers, namely, odfs 1, 5, and 6, are positively correlated with the lengths of the flagellar components. In other words, the longer is the flagellar length, the thicker is the flagellar diameter. Speculatively, longer flagella have larger mitochondrial volumes. The flagellar lengths, especially the length of the midpiece, are positively associated with the volume of mitochondria across 10 species. The sperm midpiece length was largely considered a plausible predictor of sperm swimming velocity in house mice and primates [10, 37], although some inconsistent results were reported for a passerine bird [38]. Although the reasons for this discrepancy remain unknown, our findings further support the hypothesis that sperm with longer midpieces swim faster than do sperm with shorter midpieces, which might contribute to larger mitochondrial volume and function. Notably, ATP content is slightly positively associated with sperm swimming velocity, whereas the correlation coefficient between mitochondrial volume and swimming velocity was far smaller than the correlation coefficient between mitochondrial volume and ATP content. This suggests that other unidentified factors impact swimming velocity [39]. The movement properties of the principal piece suggest that it may be a potential candidate. Unfortunately, we did not find any relationship between the area of the FS and swimming velocity or the length of any flagellar components.

Regarding sperm competition, beneficial changes in sperm morphology can increase sperm swimming velocity, which is a main determinant of fertilization success [40, 41]. For instance, a positive relationship between sperm competition levels and sperm midpiece volume has been reported by Dixson and colleagues [37]. However, the regulators of flagellar length and midpiece volume (which is equal to the sum of the mitochondrial and ODF volumes) are largely unexplored. Our study presents ODFs and/or mitochondria as potential determinants of flagellar length. The proteins involved in the development of ODFs, especially odfs 1, 5, and 6, and the size of mitochondria should be examined in further studies. Lower sperm motility, and even asthenozoospermia in humans, can occur due to deficiencies in the development of the ODFs and mitochondria [27, 42]. In fact, we found that the lengths of sperm midpieces and principal pieces from asthenospermic samples were shorter, concomitant with higher defect rates of axonemes, ODFs and mitochondrial structures, in comparison with those from normospermic samples (Additional file 8: Figure S8).

Furthermore, we found that the area of the axoneme slightly tapered from the base to the tip of the flagellum in all 10 species, in contrast to existing beliefs that the width of the axoneme is constant along flagella, even across species. To confirm this result, we detected a similar change tendency by measuring the areas of the axoneme from 46 human normospermic and 25 asthenospermic sperm samples (data not shown). In addition, slopes of axonemes from 10 species were negatively correlated with the lengths of the components of sperm flagella. In other words, the longer is the flagellar length, the smaller is the slope of the axoneme along the flagellum. The physiological functions of the inconsistent width and slopes of the axonemes across species remain unclear.

Moreover, the area of the FS was not associated with any dimension of the sperm components. Additionally, it does not seem to be the major source of ATP, although glycolysis-related kinases are located in the FS. In our opinion, the functions of the FS may focus on the signal transductions for initiating sperm movements. For example, the Catsper family of proteins, which mediate Ca^2+^ influx, are exclusively located in the FS [43]. The size of the FS may be positively correlated with the expression levels of Catsper family proteins and Ca^2+^ signal intensity during sperm capacitation [36]. In addition, the principal piece is considered to be the source of propulsion forces [44]. Speculatively, the size of the FS may be associated with the propulsion forces to mediate the swimming pattern after capacitation. This possibility should be validated in further studies.

Additionally, we did not find any relationships between the head dimension and sperm ultrastructures. In a previous study, it was reported that the head length and the head length/width ratio was associated with testes mass using data from 194 Entherian mammals [28]. Indeed, we applied these data from Eduardo RS Roldan’s work to analyse the relationships between head dimension, sperm flagellar length, and VSL. We found that the head length was slightly associated with the midpiece length and VSL; the head width was also slightly associated with the midpiece length and VSL (data not shown). Therefore, these results regarding relationships between head dimension and sperm ultrastructures may apply to few species. More mammalian species should be examined to validate our results. Furthermore, our results cannot be applied directly to resolve the relationships between flagellar lengths and sperm ultrastructures at the within-species level. For example, Tim Birkhead and colleagues found that sperm with shorter midpieces contained the highest concentration of ATP in a passerine bird, though they also found that the lengths of sperm components were important for swimming velocity [11, 38, 45], and other researchers found that faster velocities associated with higher ATP content and longer midpiece in many taxa may be due to increases in sperm competition levels [46]. Additionally, these relationships found in the present study cannot be applied to non-mammalian species. For example, the ODFs are specific accessory structures in mammalian sperm that do not exist in invertebrate sperm such as the fruit fly, fish and sea squirt [47, 48]. The longer sperm of the fruit fly (*Drosophila melanogaster*) swim more slowly than do those with relatively shorter flagella [49], though swimming velocity is unaffected by flagellar length in Atlantic salmon [50].

## Conclusion

Taken together, the common characteristics for the ten studied species are that flagella and internal ultrastructures, including MS, total Odfs, each Odf fiber, FS and axoneme, taper from the tip to end in ten species, whereas the slopes of these and shapes of sperm heads exhibit species-specific traits. Additionally, the structural description offers novel insights into the positive associations among internal structures, especially ODFs and mitochondria, and flagellar length, revealing relationships between structures and sperm physiology. Furthermore, our results not only provide original data to establish a three-dimensional view of sperm ultrastructures and morphology across 10 species at the level of electron microscopy but also highlight the potential targets. For instance, the proteins involved in the architectures of MS and ODF may also be determinants of flagellar length, which is considered to be a critical factor in sperm motility and sperm competition studies. The results of this study should enable the establishment of more precise mathematical models to better understand the development of sperm, the physics of sperm swimming and the asthenozoospermic pathogenesis.

## Additional files

Additional file 1: **Figure S1.** Relationships between areas of ODFs and outer areas across 10 species. A-J. The scatter plots show the distribution of the areas of the ODFs along flagella across 10 species. The red lines are regression lines. (JPG 1620 kb)

Additional file 2: **Figure S2.** Relationships between areas of mitochondrial sheath and outer areas across 10 species. A-J. The scatter plots show the distribution of the mitochondrial areas along flagella across 10 species. The red lines are regression lines. (JPG 1567 kb)

Additional file 3: **Figure S3.** Relationships between areas of FS and outer areas across 10 species. A-J. The scatter plots show the distribution of the areas of the FS along flagella across 10 species. The red lines are regression lines. (JPG 1619 kb)

Additional file 4: **Figure S4.** Relationships between areas of odfs 1–9 and outer areas across 10 species. A-J. The scatter plots show the distribution of the odfs 1–9 areas along flagella across 10 species. The dash lines, green lines, blue lines, pink lines, dot lines, solid lines, light blue lines, grey lines and red lines are regression lines of odfs 1–9 in species, respectively. (ZIP 2159 kb)

Additional file 5: **Figure S5.** Relationships between areas of the axonemes and outer areas across 10 species. A-J. The scatter plots show the distribution of the axoneme areas along flagella across 10 species. The red lines are regression lines. (JPG 1721 kb)

Additional file 6: **Figure S6.** Statistical results of areas of major internal ultrastructures along flagella across species. A-E. Scatter plots show the mean areas of mitochondria, FS, ODFs, axoneme and t-odfs, as well as their tendencies from positions 1 to 9. Open inverse triangles, open hexagons, solid squares, open triangles, solid circles, solid triangles, solid hexagons, open squares, solid rhombuses and open circles represent data from human, rabbit, dog, pig, guinea pig, goat, bull, mouse, golden hamster and rat, respectively. (JPG 1592 kb)

Additional file 7: **Figure S7.** Statistical results of areas of odfs 1–9 along flagella across species. A-I. Scatter plots show the mean areas of odfs 1–9 from position 1 to position 9. Open inverse triangles, open hexagons, solid squares, open triangles, solid circles, solid triangles, solid hexagons, open squares, solid rhombuses and open circles represent data from human, rabbit, dog, pig, guinea pig, goat, bull, mouse, golden hamster and rat, respectively. (ZIP 1014 kb)

Additional file 8: **Figure S8.** Comparison of sperm morphology and ultrastructures between human normozoospermic and asthenozoospermic samples. (A) The length of the midpiece in asthenozoospermic sperm is shorter than that in normozoospermic sperm. (B) The length of the principal piece in asthenozoospermic sperm is shorter than that in normozoospermic sperm. (C) The percentages of axonemes, mitochondria and ODFs with defects in asthenozoospermic samples are higher than those in normozoospermic samples. The data are presented as the means ± SEM. *N* = 46 normozoospermic samples, *N* = 25 asthenozoospermic samples, *n* ≥ 100 flagella/sample. NS, *P* > 0.05; *P < 0.05; **P < 0.01; ***P < 0.001. (JPG 1137 kb)

Additional file 9: **Table S1.** The mean parameters of the sperm morphology. (XLSX 10 kb)

Additional file 10: **Table S2.** The cross-sectional areas of the flagellar in nine positions across 10 species. (XLSX 10 kb)

Additional file 11: **Table S3.** The cross areas of ultrastructures in each sperm segment across 10 species. (XLSX 32 kb)

Additional file 12: **Table S4.** Mitochondrial volumes, ATP content and VSL across 10 species. (XLSX 10 kb)

Additional file 13: **Table S5.** Regression correlation analysis data of the sperm ultrastuctures across 10 species. (XLSX 20 kb)

Additional file 14: **Table S6.** The parameters of asthenozoospermic and normozoospermic semen samples. (XLSX 9 kb)

## Data Availability

All data generated or analysed during this study are included in this published article [and its supplementary information files].

## Abbreviations

FS: Fibrous sheath
IPMCH: International Peace Maternity & Child Health Hospital
MS: Mitochondrial sheath
ODFs: Outer dense fibers
VSL: Velocity of straight-line
