# Supplementary figures and images for: Comparative analysis of mammalian sperm ultrastructure reveals relationships between sperm morphology, mitochondrial functions and motility
Source: Reprod Biol Endocrinol. 2019 Aug 15;17:66. doi: 10.1186/s12958-019-0510-y (PMC6696699; doi:10.1186/s12958-019-0510-y)

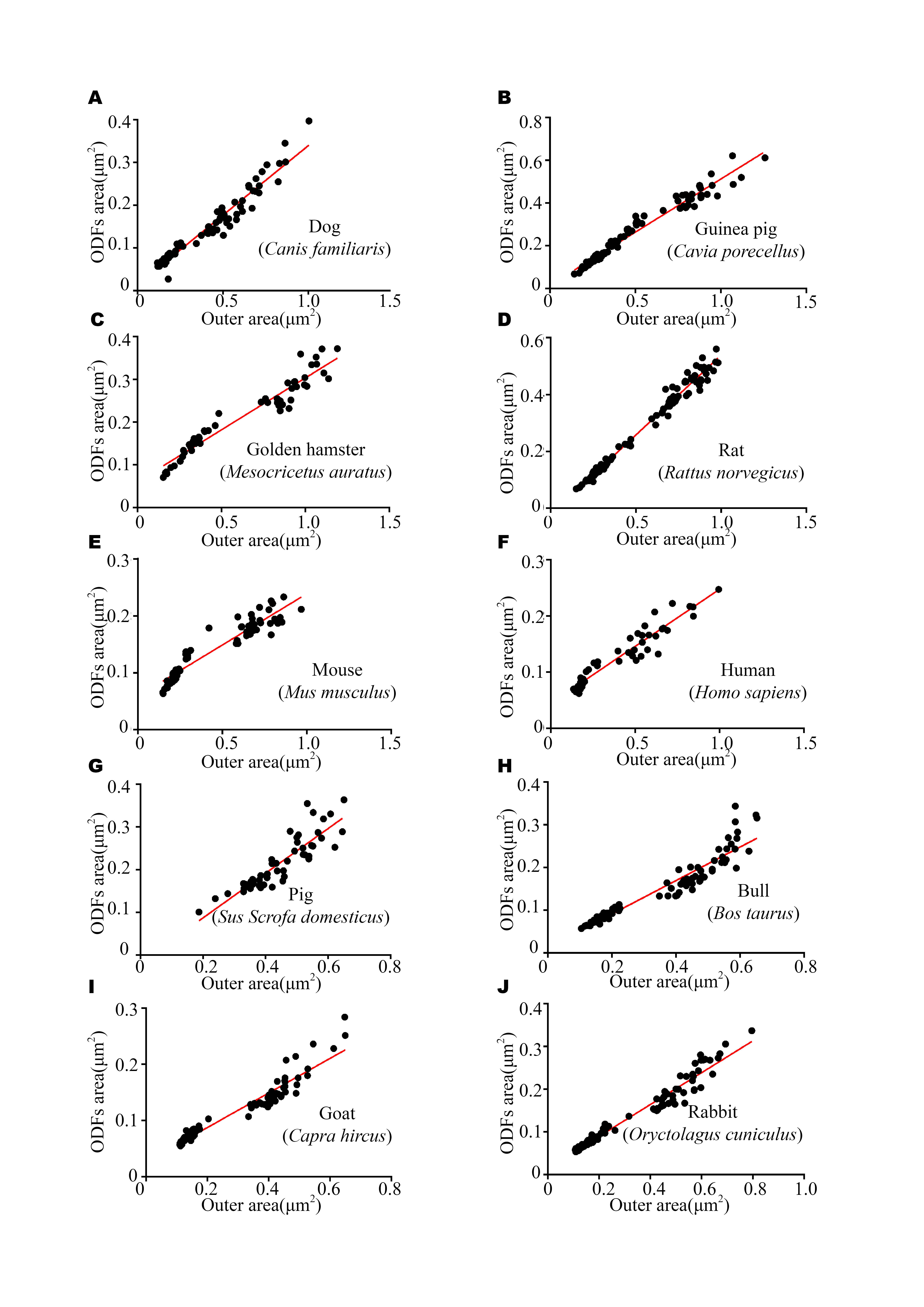

Supplement: Supplementary file 1 — Figure S1. Relationships between areas of ODFs and outer areas across 10 species. A-J. The scatter plots show the distribution of the areas of the ODFs along flagella across 10 species. The red lines are regression lines. (JPG 1620 kb) [file 12958_2019_510_MOESM1_ESM.jpg]

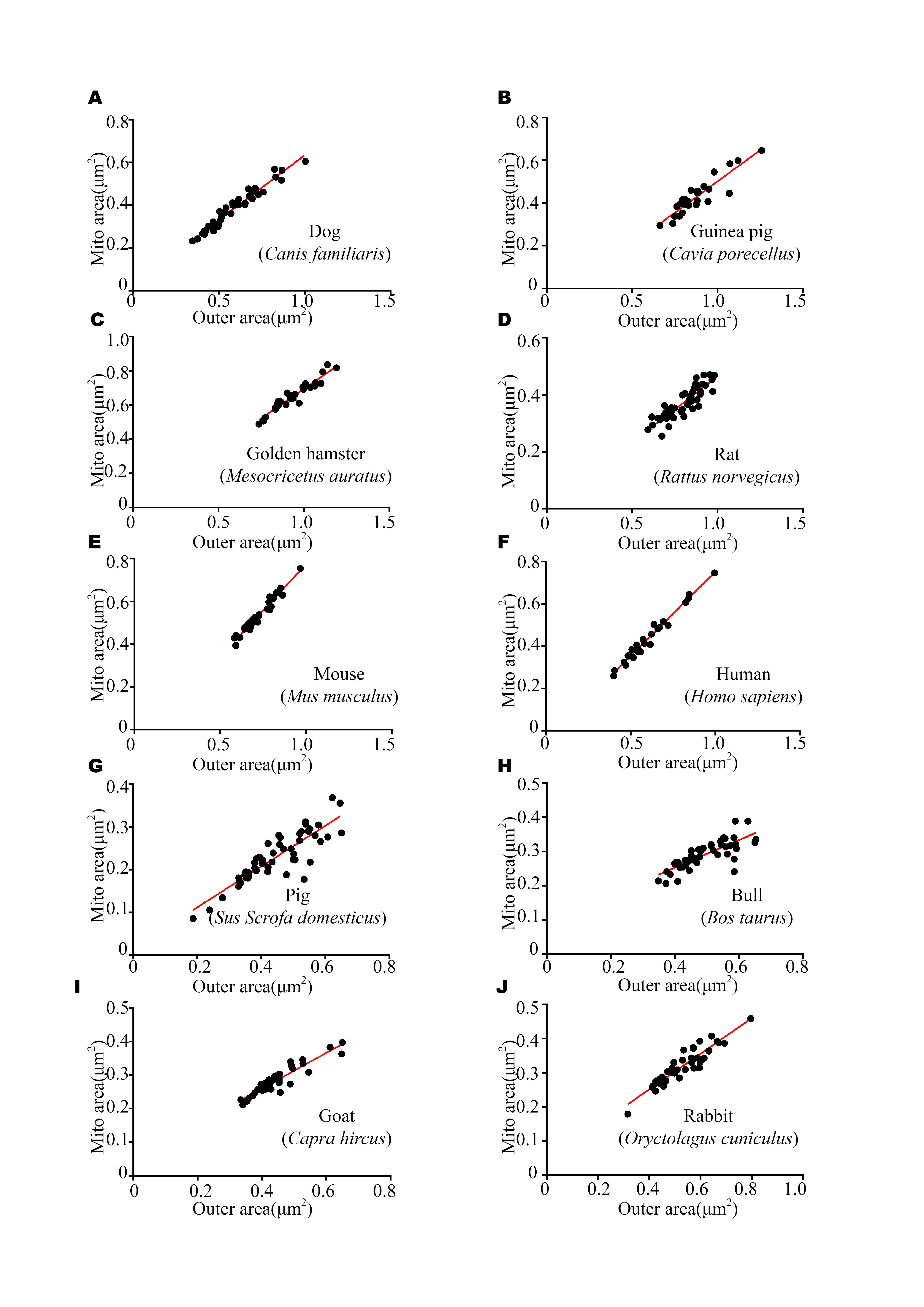

Supplement: Supplementary file 2 — Figure S2. Relationships between areas of mitochondrial sheath and outer areas across 10 species. A-J. The scatter plots show the distribution of the mitochondrial areas along flagella across 10 species. The red lines are regression lines. (JPG 1567 kb) [file 12958_2019_510_MOESM2_ESM.jpg]

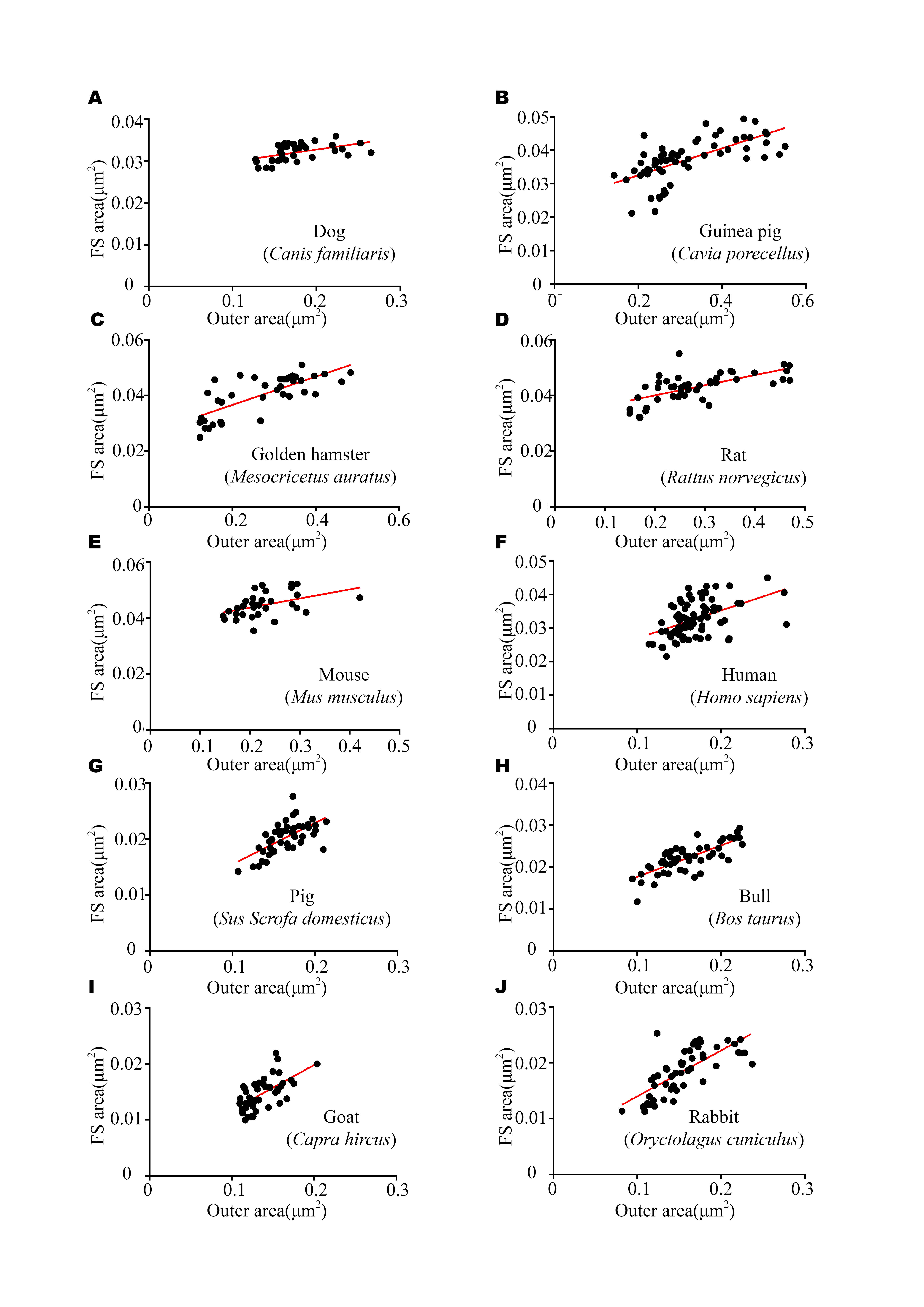

Supplement: Supplementary file 3 — Figure S3. Relationships between areas of FS and outer areas across 10 species. A-J. The scatter plots show the distribution of the areas of the FS along flagella across 10 species. The red lines are regression lines. (JPG 1619 kb) [file 12958_2019_510_MOESM3_ESM.jpg]

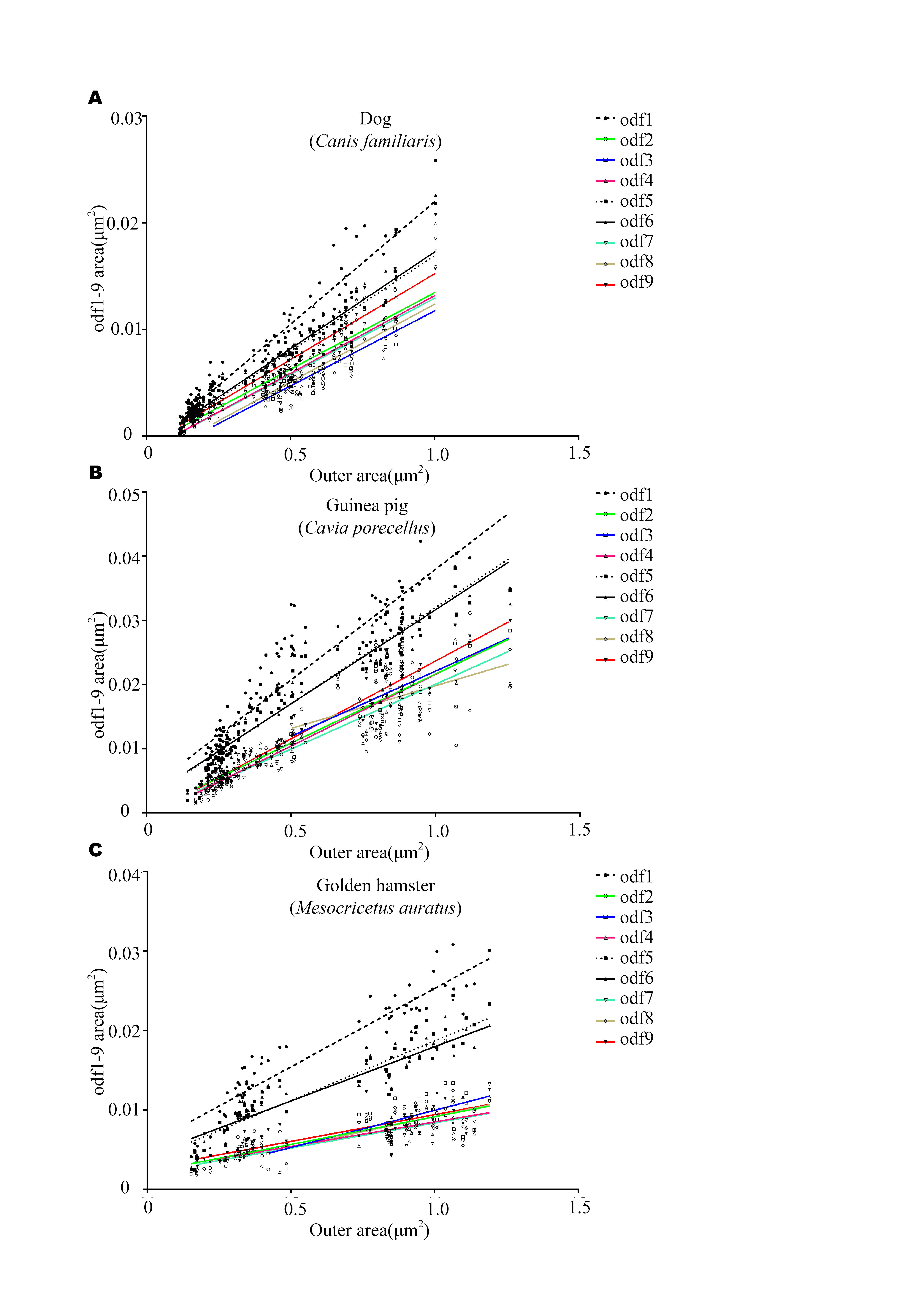

Supplement: Supplementary file 4 — Figure S4. Relationships between areas of odfs 1–9 and outer areas across 10 species. A-J. The scatter plots show the distribution of the odfs 1–9 areas along flagella across 10 species. The dash lines, green lines, blue lines, pink lines, dot lines, solid lines, light blue lines, grey lines and red lines are regression lines of odfs 1–9 in species, respectively. (ZIP 2159 kb) [file 12958_2019_510_MOESM4_ESM.zip › fig S4a Odf1-9 area and outer area-revised.jpg]

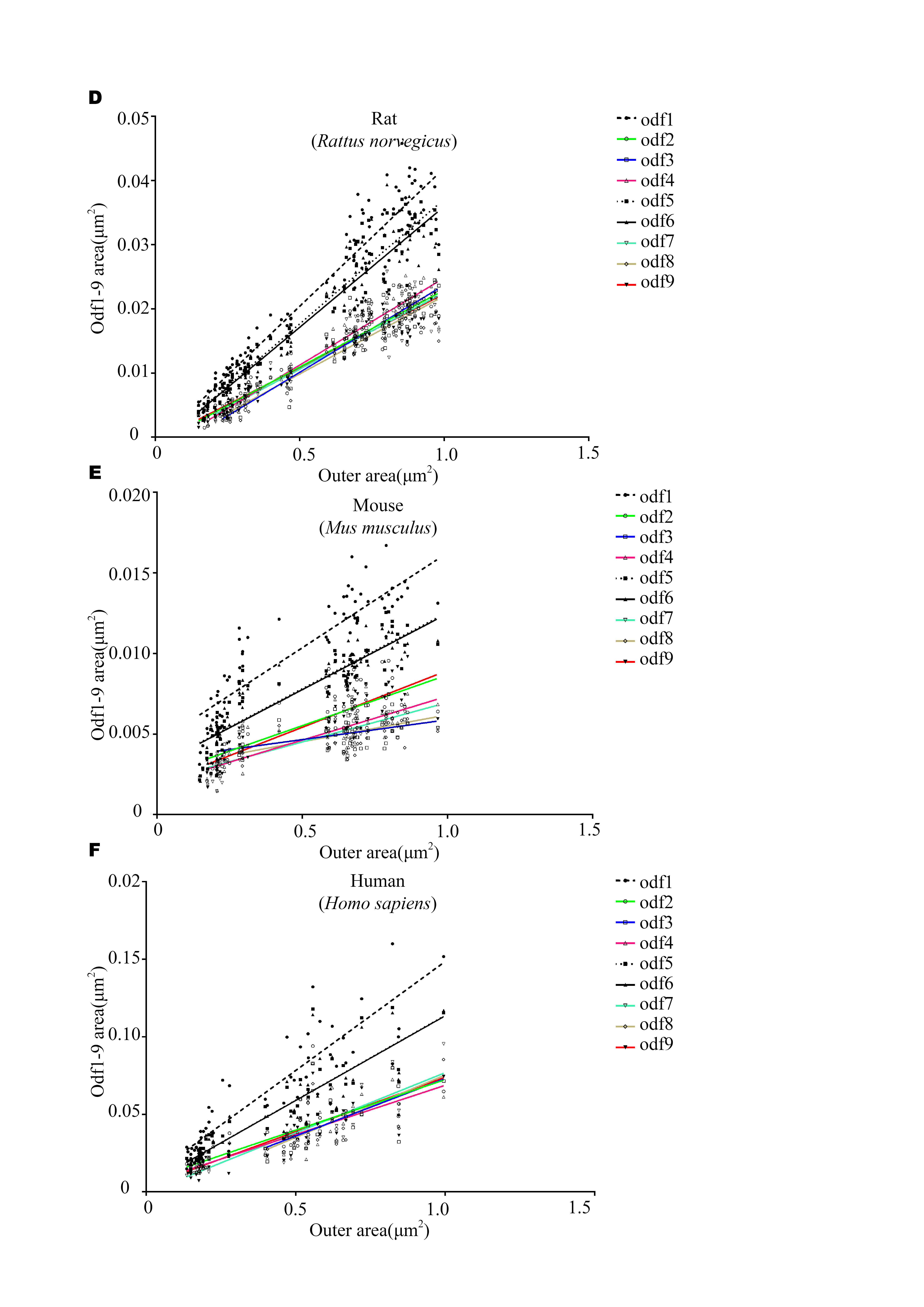

Supplement: Supplementary file 4 — Figure S4. Relationships between areas of odfs 1–9 and outer areas across 10 species. A-J. The scatter plots show the distribution of the odfs 1–9 areas along flagella across 10 species. The dash lines, green lines, blue lines, pink lines, dot lines, solid lines, light blue lines, grey lines and red lines are regression lines of odfs 1–9 in species, respectively. (ZIP 2159 kb) [file 12958_2019_510_MOESM4_ESM.zip › fig S4b Odf1-9 area and outer area-revised.jpg]

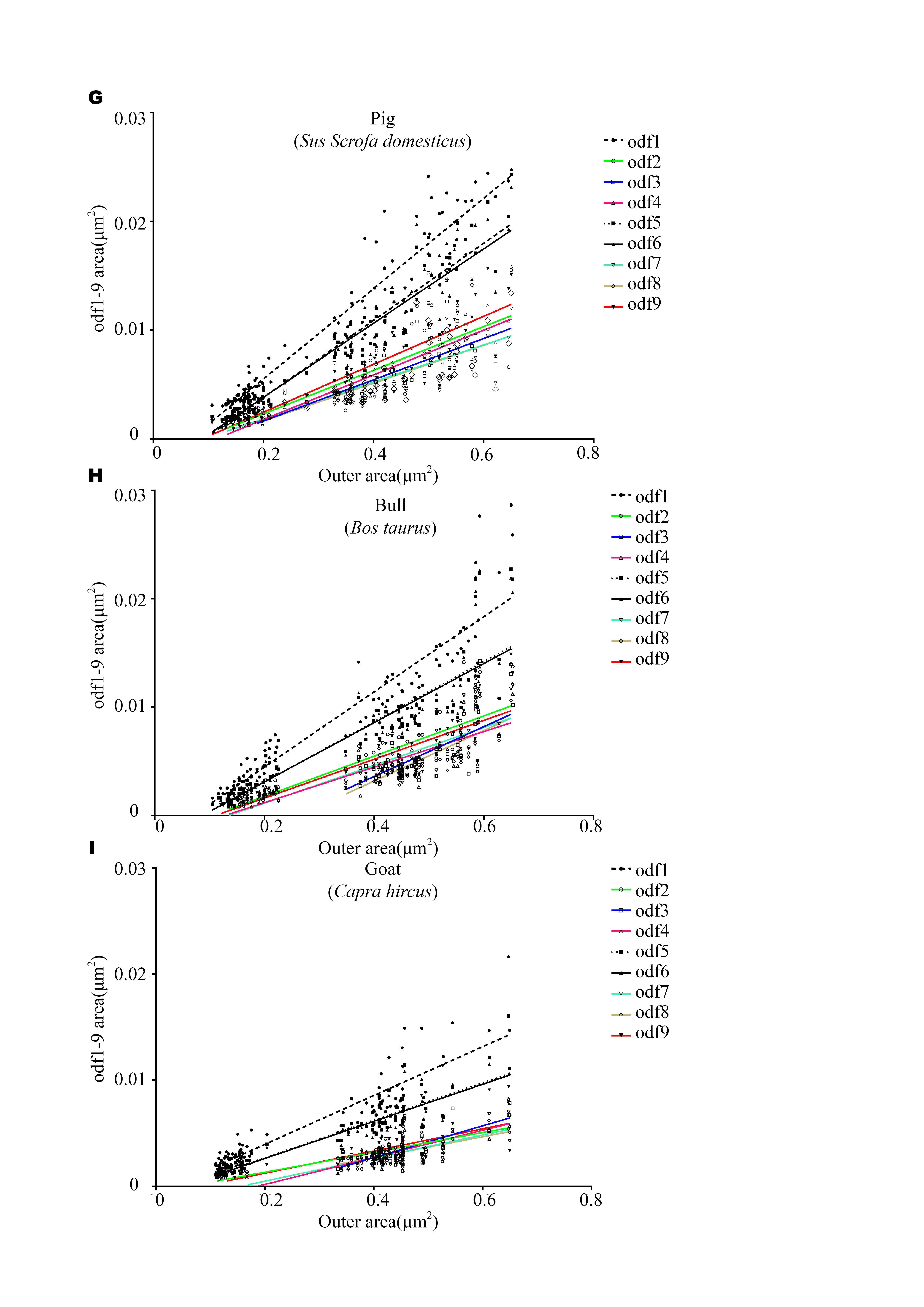

Supplement: Supplementary file 4 — Figure S4. Relationships between areas of odfs 1–9 and outer areas across 10 species. A-J. The scatter plots show the distribution of the odfs 1–9 areas along flagella across 10 species. The dash lines, green lines, blue lines, pink lines, dot lines, solid lines, light blue lines, grey lines and red lines are regression lines of odfs 1–9 in species, respectively. (ZIP 2159 kb) [file 12958_2019_510_MOESM4_ESM.zip › fig S4c Odf1-9 area and outer area-revised.jpg]

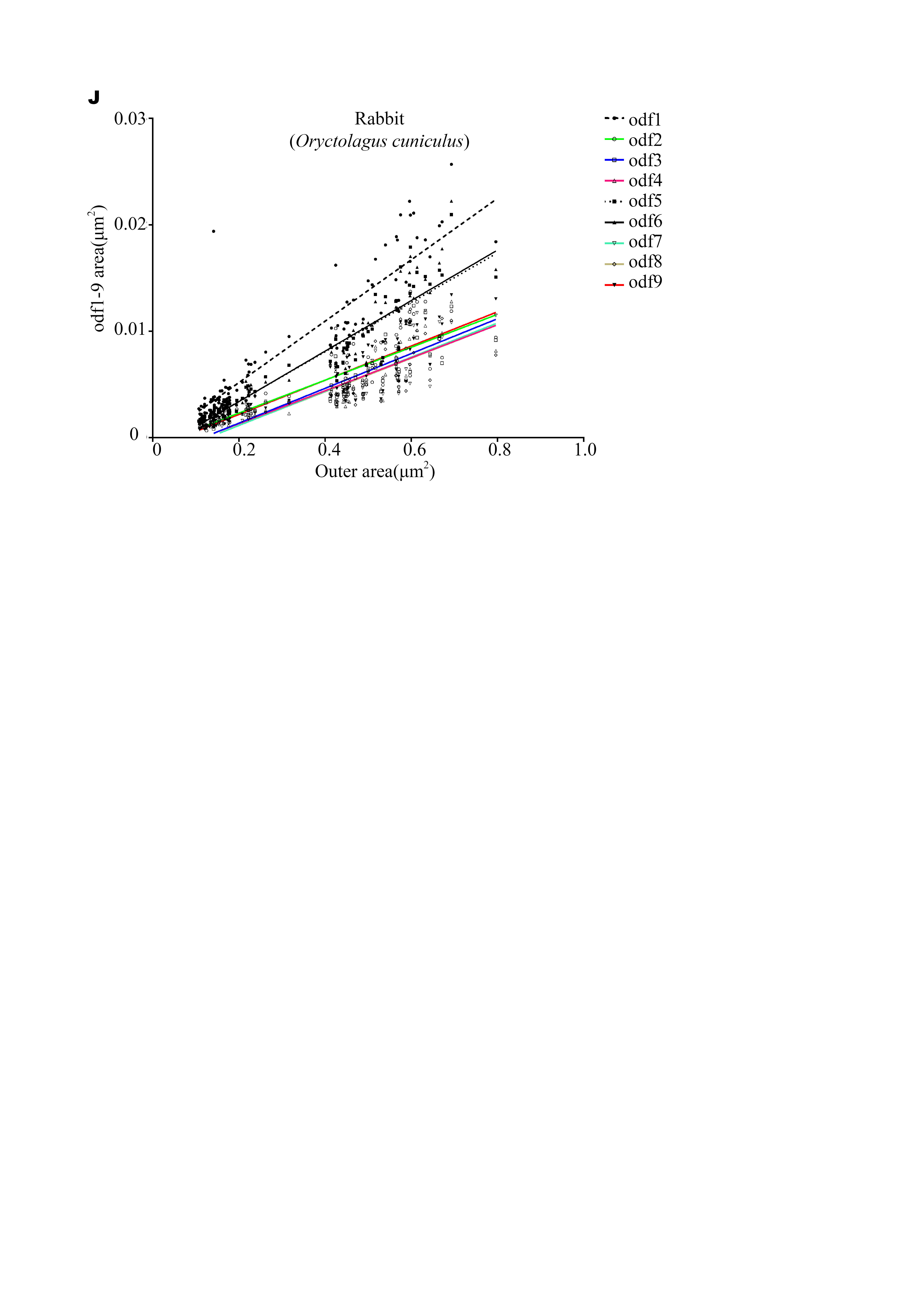

Supplement: Supplementary file 4 — Figure S4. Relationships between areas of odfs 1–9 and outer areas across 10 species. A-J. The scatter plots show the distribution of the odfs 1–9 areas along flagella across 10 species. The dash lines, green lines, blue lines, pink lines, dot lines, solid lines, light blue lines, grey lines and red lines are regression lines of odfs 1–9 in species, respectively. (ZIP 2159 kb) [file 12958_2019_510_MOESM4_ESM.zip › fig S4d Odf1-9 area and outer area-revised.jpg]

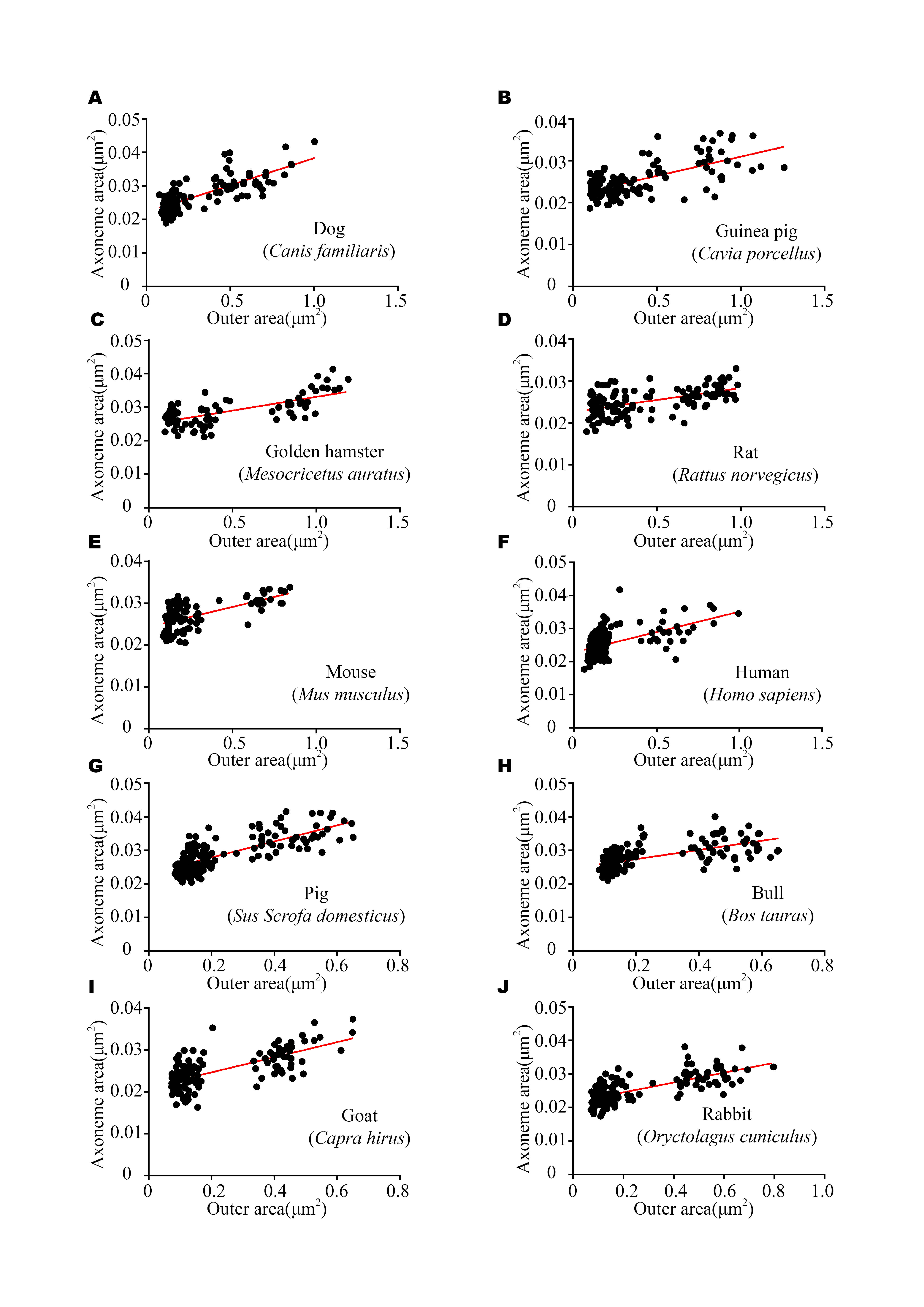

Supplement: Supplementary file 5 — Figure S5. Relationships between areas of the axonemes and outer areas across 10 species. A-J. The scatter plots show the distribution of the axoneme areas along flagella across 10 species. The red lines are regression lines. (JPG 1721 kb) [file 12958_2019_510_MOESM5_ESM.jpg]

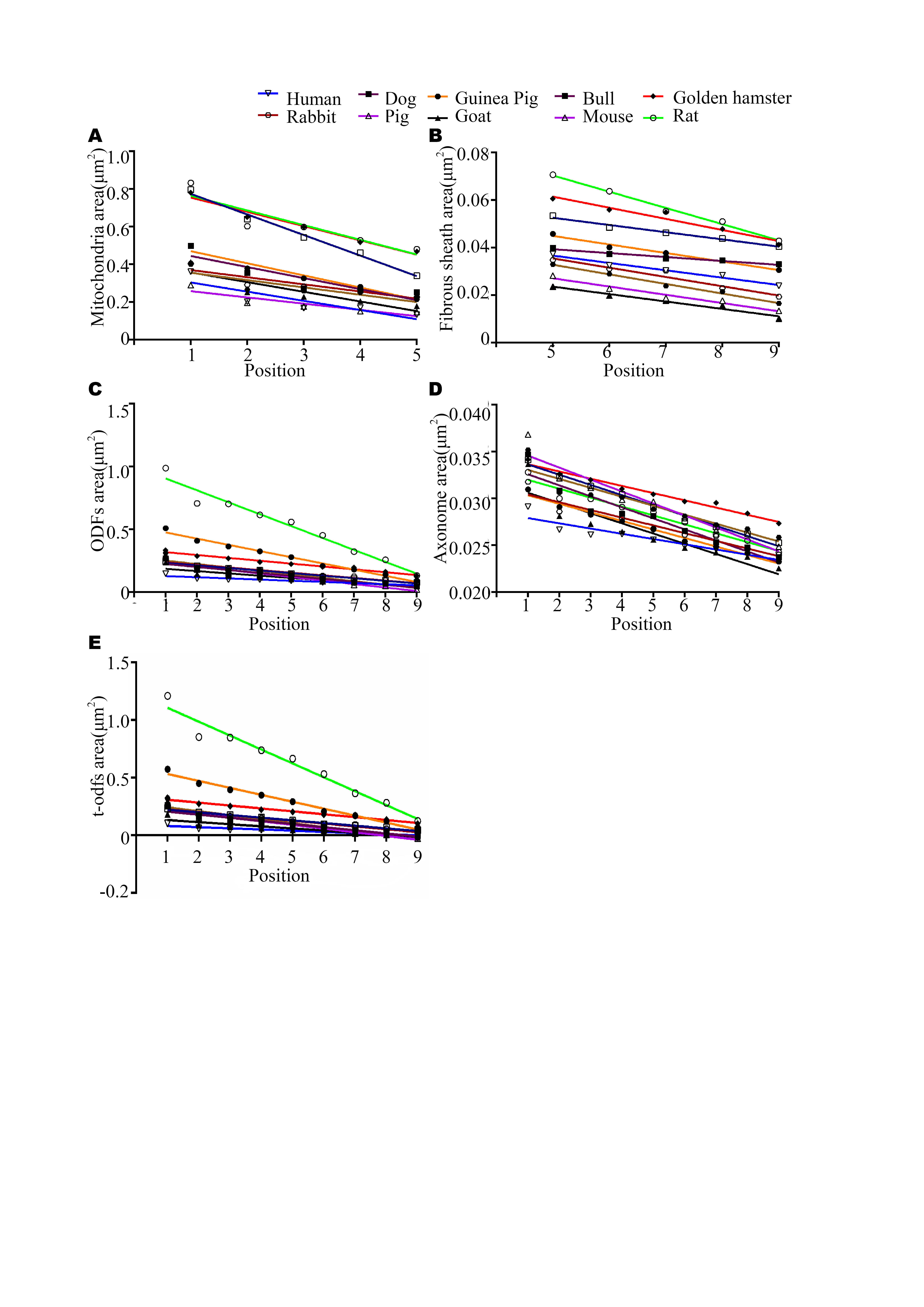

Supplement: Supplementary file 6 — Figure S6. Statistical results of areas of major internal ultrastructures along flagella across species. A-E. Scatter plots show the mean areas of mitochondria, FS, ODFs, axoneme and t-odfs, as well as their tendencies from positions 1 to 9. Open inverse triangles, open hexagons, solid squares, open triangles, solid circles, solid triangles, solid hexagons, open squares, solid rhombuses and open circles represent data from human, rabbit, dog, pig, guinea pig, goat, bull, mouse, golden hamster and rat, respectively. (JPG 1592 kb) [file 12958_2019_510_MOESM6_ESM.jpg]

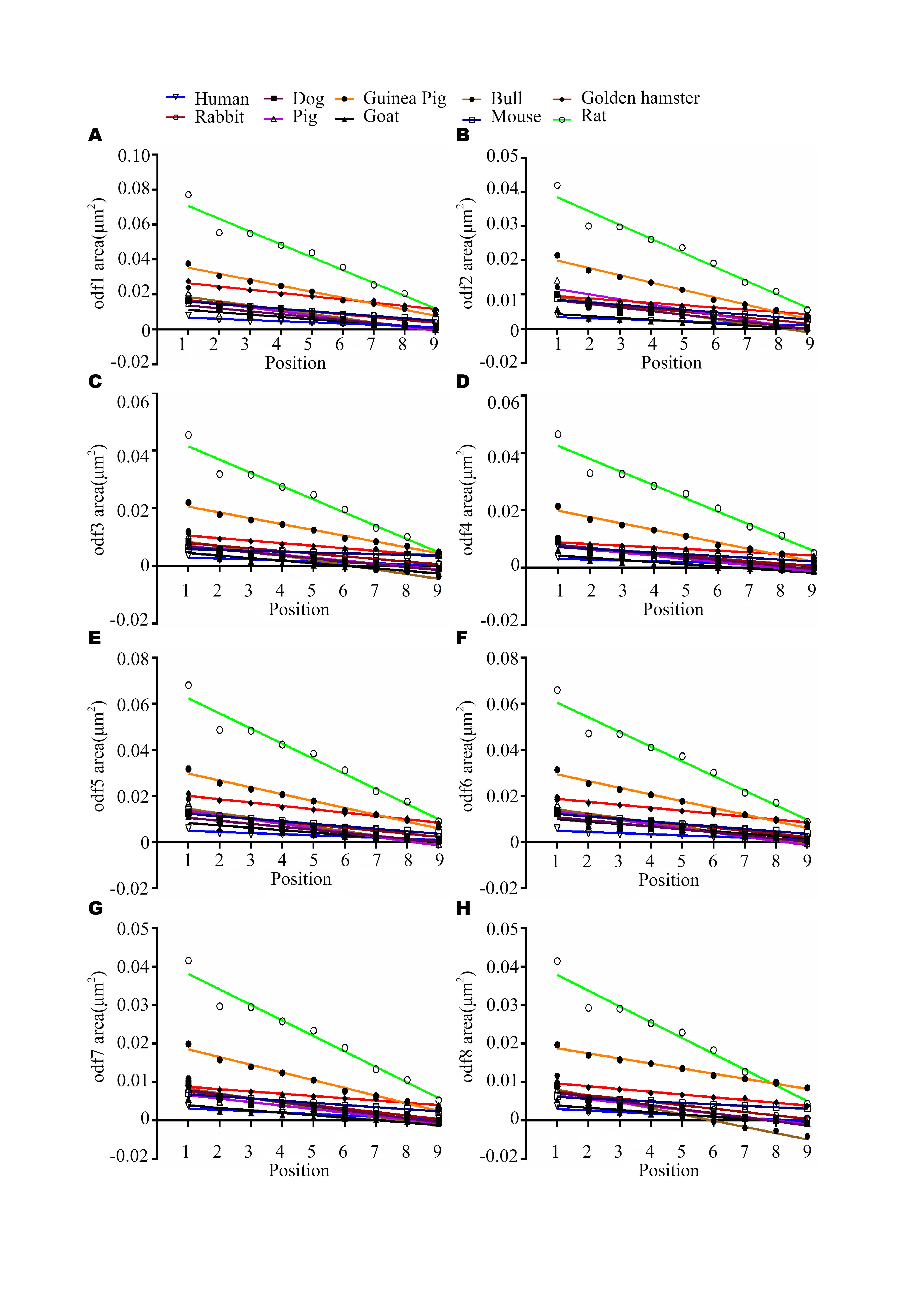

Supplement: Supplementary file 7 — Figure S7. Statistical results of areas of odfs 1–9 along flagella across species. A-I. Scatter plots show the mean areas of odfs 1–9 from position 1 to position 9. Open inverse triangles, open hexagons, solid squares, open triangles, solid circles, solid triangles, solid hexagons, open squares, solid rhombuses and open circles represent data from human, rabbit, dog, pig, guinea pig, goat, bull, mouse, golden hamster and rat, respectively. (ZIP 1014 kb) [file 12958_2019_510_MOESM7_ESM.zip › fig S7a Odf1-9 area.jpg]

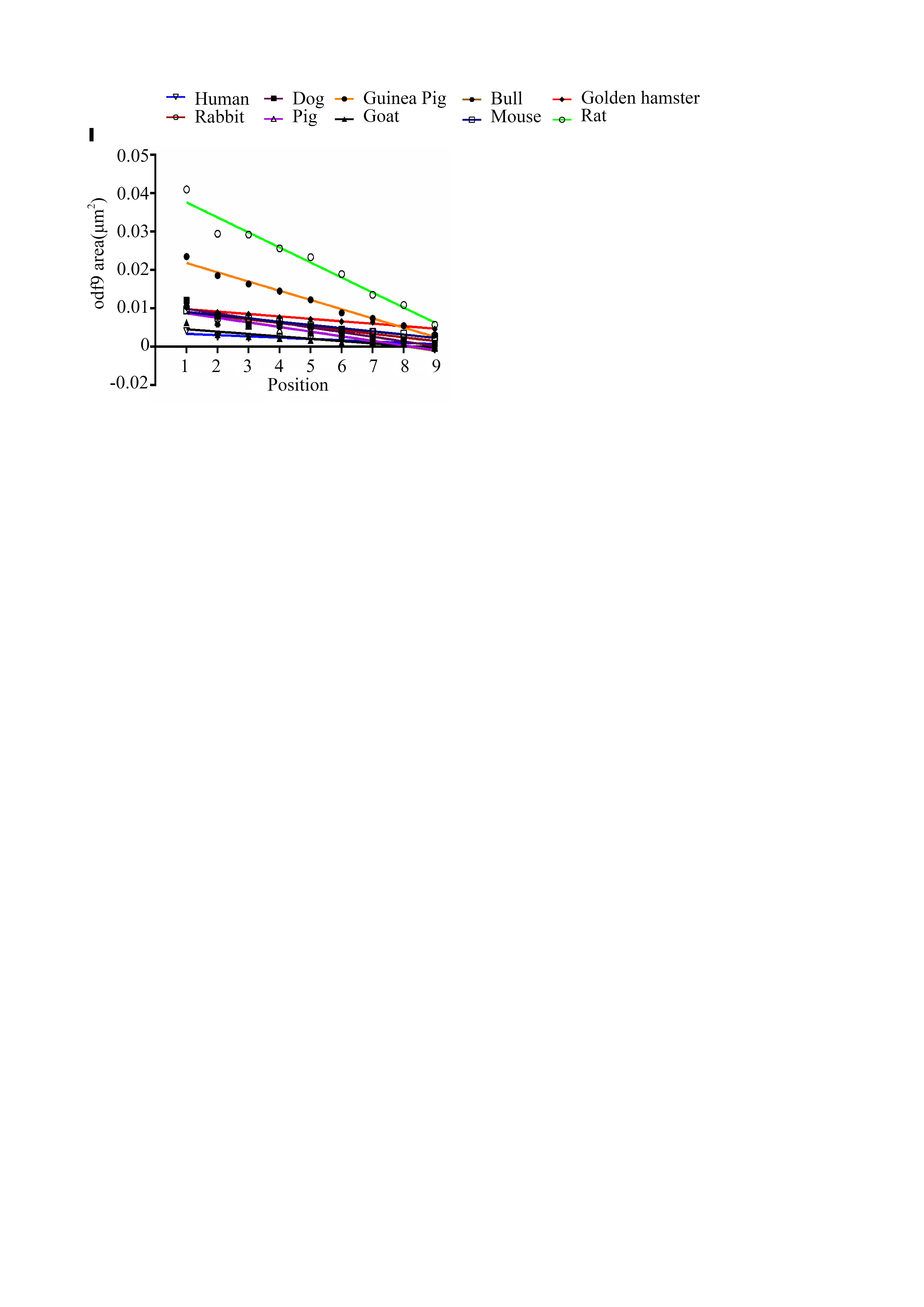

Supplement: Supplementary file 7 — Figure S7. Statistical results of areas of odfs 1–9 along flagella across species. A-I. Scatter plots show the mean areas of odfs 1–9 from position 1 to position 9. Open inverse triangles, open hexagons, solid squares, open triangles, solid circles, solid triangles, solid hexagons, open squares, solid rhombuses and open circles represent data from human, rabbit, dog, pig, guinea pig, goat, bull, mouse, golden hamster and rat, respectively. (ZIP 1014 kb) [file 12958_2019_510_MOESM7_ESM.zip › fig S7b Odf1-9 area.jpg]

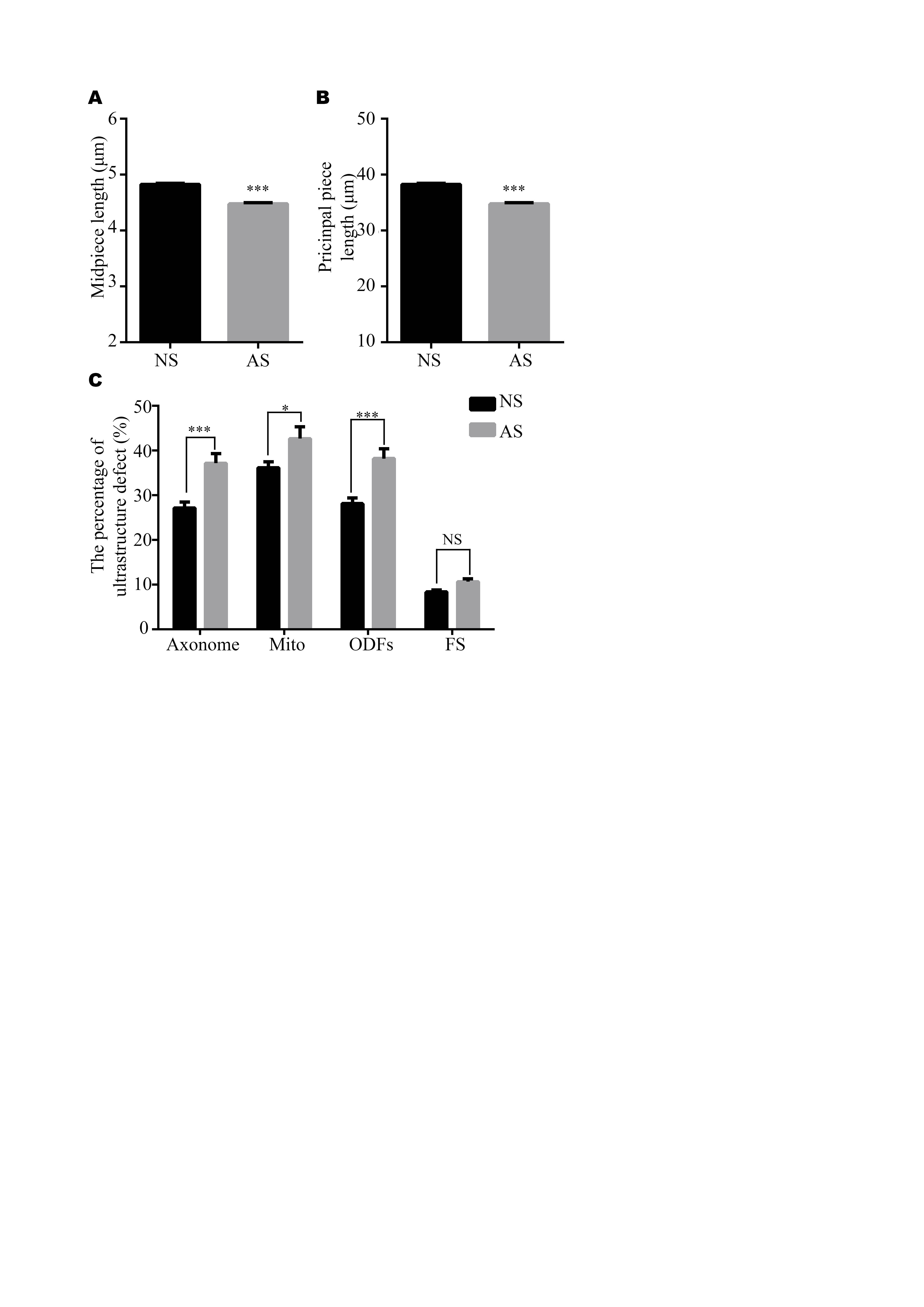

Supplement: Supplementary file 8 — Figure S8. Comparison of sperm morphology and ultrastructures between human normozoospermic and asthenozoospermic samples. (A) The length of the midpiece in asthenozoospermic sperm is shorter than that in normozoospermic sperm. (B) The length of the principal piece in asthenozoospermic sperm is shorter than that in normozoospermic sperm. (C) The percentages of axonemes, mitochondria and ODFs with defects in asthenozoospermic samples are higher than those in normozoospermic samples. The data are presented as the means ± SEM. N = 46 normozoospermic samples, N = 25 asthenozoospermic samples, n ≥ 100 flagella/sample. NS, P > 0.05; *P < 0.05; **P < 0.01; ***P < 0.001. (JPG 1137 kb) [file 12958_2019_510_MOESM8_ESM.jpg]
